# Therapeutics of Convulsions

**Published:** 1889-12

**Authors:** H. P. Holmes

**Affiliations:** Oak Park, Ill.


### THERAPEUTICS OF CONVULSIONS.

H. P. Holmes, M. D., Oak Park, Ill.

Convulsions of Infants: Acon., Æthusa-cyn., Agar., Aml-nit., Apis-mel., Arn., Ars., Bell., Bry., Camph., Caust., Cham., Cic., Cim., Cina, Coff., Cup., Gels., Hell., Hyos., Ign., Hepar, Ipec., Kreos., Lauro., Lyc., Melilot., Merc., Nux, Opi., Sil., Stan., Stram., Tart.-emet., Tereb., Verat.-alb., Zinc.

Puerperal Convulsions: Acon., Argent.-nit., Arn., Bell., Canth., Cham., Cic., Cocc., Cup., Gels., Glon., Hell., Hyos., Ign., Lach., Lauro., Merc., Mosch., Nux-m., Œnanthe, Opi., Puls., Sec., Stram., Verat-vir., Zinc.

Epilepsy: Agar., Amyl-nit., Argent-nit., Bell., Bufo., Calc-carb., Camph., Cann-ind., Caust., Cic.-vir., Crotal., Cup., Cypri., Glon., Hydr-ac., Hyos., Ign., Indi., Kali-brom., Lach., Nux-vom., Opi., Œnanthe-croc., Plumb., Sil., Stann., Stram., Sul., Tarent., Visc-alb., Zinc., Zizea-aur.

Catalepsy: If caused by anger and vexation, Cham., Bry.; if caused by fright, Acon., Bell., Ign., Gels., Op.; if by sudden joy, Coffea; if by grief, Ign., Phos.-ac., Staph.; if by jealousy, Hyos., Lach.; if by sexual erethism, Con., Plat., Stram.; if by disappointed love, Ign., Hyos., Lach.; if by religious excitement, Stram., Sulph., Verat-alb.; in consequence of onanism, China, Nux-vom.

Aconite.—Convulsions, when in their incipiency there is great febrile excitement; hot, dry skin; restlessness, anxiety, anguish, fear of death; more or less cerebral congestion; twitchings of single muscles; child gnaws its fists, frets, cries; costive or dark watery stools; vertigo on rising from a recumbent position; dreads too much activity about her.

ÆTHUSA-CYN.—Spasms, with stupor, delirium; turning of the eyes downward; epileptiform spasms, with clenched thumbs, red face; dilating, starting, immovable pupils; foam at the mouth; teeth set; pulse small, hard, accelerated; great weakness; children cannot stand, or hold up the head.

AGARICUS.—Spasms, with tremors of the body; involuntary movements while awake; epilepsy with great exertion of strength; from fright; every seven days; from suppressed eruption; epileptic fits increase at first and lessen gradually; patient feels as if drunken and always sleepy; unconscious and speechless with the convulsions, face blue and puffed, froth at the mouth; sensation as if cold air was spreading from the spine over the body, like an *aura epileptica.*

AMYL-NITRITE.—Convulsions, with unconsciousness and inability to swallow; frequent piercing shrieks; after long-continued convulsions, weak, emaciation with tendency to sweat easily from slight exertion; during convulsions rigidity of muscles of limbs; epilepsy; muscular twitching in arms, legs, and face, followed by sense of fullness of head, flushing of face, violent palpitation of heart, and unconsciousness; mental confusion, and a dream-like state; haunted many times a day by an indescribable dread and sensation of an oncoming fit; profound and repeated yawning during unconsciousness; succession of fits with increasing frequency, before one fit ceases another one begins.

APIS-MEL.—Nervous restlessness; convulsions; trembling and jerking of the limbs; shrieking; boring the head in the pillow; cerebral affections.

ARGENTUM-NITR.—Puerperal convulsions; she has a presentment of the approaching spasm; she is in constant motion from the time she comes out of one spasm until she goes into another; the spasms are violent and are preceded by a sensation of expansion of the whole body, especially of the face and head; sometimes the woman lies quietly for some time after the spasm has ceased, but becomes very restless before another begins; cerebral epilepsy; the pupils permanently dilated a day or two before the fit; epileptic convulsions coming on at night.

ARNICA.—Where the spasm arises in consequence of a fall or other injury; pulse full and strong, and during every pain the blood rushes violently to the face and head; symptoms of paralysis of the left side; tympanitis of abdomen after labor (peritonitis); unconsciousness; involuntary discharges of fæces and urine; while the head is very hot the body is cool.

ARSENICUM.—The child lies as if dead; pale but warm; is breathless for some time; finally it twists its mouth to one side then to the other; a violent jerk appears to pass through the whole body, and its respiration and consciousness gradually return; the spasms return at longer or shorter intervals; worse the latter part of the night.

BELLADONNA.—Convulsions; starting from sleep with a wild look, dilated pupils; heat of the head and hands; red eyes and flushed face; sopor after the spasm; convulsions may commence in an arm, and then the body be thrown forward and backward; jerking and twitching of muscles between the spasms; convulsive movements in the limbs and muscles of the face; paralysis of right side of tongue; loss of speech and difficult deglutition; renewal of the fits at every pain; more or less tossing between the spasms or deep sleep, with grimaces or starts and cries, with fearful visions; sound sleep or unconsciousness after a spasm; moaning at night, even without much sleep; sleepiness, but cannot go to sleep; wild look; fresh cases of epilepsy, with decided brain symptoms; there is an aura as if a mouse were running over an extremity, or of heat rising from the stomach, or illusions of sight or of hearing; convulsions commence in upper extremities, and extend to the mouth, face, and eyes; spasm in the larynx and fauces, with a peculiar clutching of the throat during the fit; inability to swallow and danger of suffocation; foam at the mouth; involuntary micturition and defecation; oppression of the chest and anxious breathing; the spasms are excited by the least touch; great anxiety, fear, frightful visions.

BRYONIA.—Spasms developed through repercussion of the measles; dry, parched lips; patient cannot bear to be moved.

BUFO.—Epilepsy following onanism; longs for solitude to give himself up to his vice; quick ejaculation without pleasure, with spasms and painful weariness of the limbs; epilepsy, with destructive suppuration; fits occur mostly at the change of the moon, at the time of menses, in sleep.

CALCAREA-CARB.—Hemorrhoidal plethoric constitution; scrofulous and rachitic; delicate and poorly-nourished persons; sudden attacks of vertigo; loss of consciousness without convulsions; pharyngeal spasms, followed by desire to swallow; attacks return after the slightest vexation; anxiety with the fit, and after it apprehension of never getting well again; mental dullness or even derangement; speechless; nocturnal epilepsy; attack commences in abdomen.

CAMPHOR.—Spasms from suppressed catarrh of the head or chest; epileptic fits, with stertorous breathing, red and bloated face, coma; early enough given it may prevent the fit or at least abridge its intensity and duration.

CANNABIS-IND.—Epilepsy; extraordinary mental and physical vigor, an ecstatic exaltation of all the powers of mind and body as the aura before the fit; tendency to catalepsy.

CANTHARIS.—Convulsions, with dysuria and hydrophobic symptoms; bright light, drink, sound of falling water, or the mere touch of the larynx causes a renewal of the spasms.

CAULOPHYLLUM.—Convulsions, with very weak and irregular labor pains; she feels very weak.

CAUSTICUM.—Convulsive motions of the upper part of the body, with feverish heat and coldness of the hands and feet; convulsions of the extremities in the evening when the child is sleeping, with disturbed eyes and icy coldness of the body; when the paroxysms are complicated with screams, gnashing of the teeth, and violent movements of the limbs, etc.; epilepsy; recent and light cases; sudden fall, with loss of consciousness in the fresh air, but recovers himself soon; convulsions, especially on the right side, with drawing of the head toward it; during the convulsions the urine flows copiously and involuntarily; frequent urination; restless, desire to escape; idiotic condition before the attack; cold water brings the paroxysms on again.

CHAMOMILLA.—Child makes itself stiff and bends backward; kicks with the feet and screams immoderately; convulsions of children; legs moved up and down; grasping and reaching with the hands; mouth drawn from side to side; the eyes staring, jerking and twitching even in sleep; convulsions in the child caused by a fit of anger in the nurse; convulsions after anger; excessive irritability and petulance; one cheek red, the other pale; starts and shocks during sleep.

CHINA.—The loss of a large quantity of blood is the exciting cause of the eclampsia.

CICUTA-VIR.—Violent shocks through the head, arms, and legs, which cause them to jerk suddenly; spasmodic rigidity of the body, either opisthotonos or emprosthotonos; the child seems well and in great spirits, when suddenly it becomes rigid, then relaxation sets in, with great prostration; tonic spasms, renewed from the slightest touch, or the least talking or walking about; helminthiasis; strange contortions of the upper part of the body and limbs during the paroxsyms, with blue face and frequent interruptions of breathing for a few seconds; epilepsy; especially for children; convulsions; clonic and tonic spasms, with pale and yellowish complexion; distortions of the extremities; cries; frothy saliva; after the attack the child is unconscious and nearly lifeless; in women, after emotions, with sad melancholy; after confinements; painful ulcers on edge of tongue.

CIMICIFUGA.—Children wake at night with a frightened look and trembling of the limbs.

CINA.—Child is feeble, lax, and ailing; painful sensibility in the limbs of whole body on motion or touch; attack worse early in morning and evening, and most violent after eating; convulsive attacks at night; spasms of children, with throwing the arms from side to side; convulsions of the extensor muscles, the child becomes suddenly stiff, followed by trembling of the whole body, with blue lips, and whining complaints of pain in throat, chest, and all the limbs; there is a clucking noise during convulsion as if water was poured out of a bottle, from the throat down to abdomen, paralytic pains in arms and legs; the child exhibits vermiculous symptoms; discharges worms; hacking cough; continually making attempts at deglutition, as if to swallow something down; is very difficult to be pleased with anything.

COFFEA.—Convulsions of teething children, with grinding of teeth and coldness of limbs, after over-excitement; the attack has been brought on by excessive laughing and playing; weakly and excitable children; and in consequence frequently suffers with spasms.

CROTALIS-HOR.—Convulsions, with trembling of the limbs, without foaming at the mouth; loss of senses; indifference, seems only half alive; paleness of the face, as in faintness; sensation of tight constriction of the throat.

CUPRUM.—Eclampsia of children during dentition; the spasm is often preceded by violent vomiting of phlegm; the clonic spasms begin in the fingers and toes; child lies on belly and spasmodically thrusts the breach up; after the convulsion the child screams, and turns and twists in all directions until another spasm occurs; opisthotonos with every paroxysm, with spreading out of the limbs and opening of the mouth; clonic spasms during pregnancy, when the attack begins at the periphery and extends centrally; nocturnal epilepsy, or when the fits return at regular intervals [menses], beginning with a sudden scream; unconsciousness; loss of sensibility and throwing the body upward and forward; convulsions commencing at the fingers or toes, or in the arms, with coldness of the hands and feet, and pallor or lividity of face; clenching the thumbs; suffocating paroxysms; frequent emission of urine; piercing, violent screaming; difficult comprehension or screaming; convulsion of children during dentition or from retrocession of an exanthema; extreme violence of the convulsion with pale or livid face, slow pulse [often a sign of feeble muscular action of the heart], coldness of hands and feet.

CUPRUM-ACET.—Spasms from retrocession of the eruption in scarlet fever.

CYPRIPEDIUM.—Epilepsy from reflex nervous irritation; from exhaustion of nerve-forces; from irritability of the brain in children.

GELSEMINUM.—Convulsions from reflex irritation; premonitory symptoms; the head feels very large; the spasms occur as the first hint that the os uteri remains rigid and unchanged; distressing pains from before backward and upward in the abdomen; heavy head, with half stupid look; face deep red; speech thick; pulse slow, full; albuminuria.

GLONOINE.—Epileptic fits accumulate and return daily; convulsions from cerebral congestion; stupidity and somnolence; alternate congestion of heart and head; throbbing pain in the epigastrium.

HEPAR-SULPH.—Traumatic convulsions, caused by excessive pressure on the brain during delivery; trismus of new-born babes.

HELLEBORUS.—Convulsions of nursing children, with extreme coldness; the urine is very dark, and has a sediment like coffee-grounds; intense and intolerable pains in the head; a shock passes through the brain as if from electricity, followed by spasms.

HYDROCYANIC ACID.—When the muscles of the back, face, and jaws are principally affected, and the body assumes a bluish tint; epilepsy; recent cases; sudden complete loss of consciousness and sensation; extreme coma for several hours, only interrupted by occasional sudden convulsive movements; confusion of the head and vertigo; jaws clenched, teeth firmly set, froth at the mouth, foaming large bubbles; unable to swallow; involuntary discharge of urine and fæces; upper extremities contracted and the hands clenched; unusual stiffness of the legs; spasms commencing in the toes, followed by distortion of the eyes, toward the right and upward, afterward general spasms; distortion of the limbs and frightful distortion of the face; trunk spasmodically bent forward; great exhaustion, prostration and aversion to all work, mental or physical.

HYDROPHOBIN.—The spasms are excited whenever she attempts to drink water, or if she hears it pouring from one vessel into another; the remedy may also be indicated by the sight or sound of water affecting the patient unpleasantly, even though she desires water.

Hyoscyamus.—Convulsions after meals; the child sickens after eating, vomits or shows distress at the stomach; sudden shrieks and then insensible; convulsive jerks; long-lasting spasms; frothing at the mouth; every muscle in the body is convulsed—the eyes, the eye-lids, the muscles of the face; puerperal spasms; shrieks; anguish; oppression of the chest; unconsciousness; bluish color of the face; twitching and jactitation of every muscle of the body; delirium; during convulsion limbs forcibly curved and thrown up from the bed; epilepsy, before the fit, vertigo; sparks before the eyes; ringing in the ears; hungry gnawing; during the fit face purple, eyes projecting, shrieks, grinding teeth, urination; after attack, sopor, snoring; from grief, after emotion.

Ignatia.—Spasms return at the same hour every day; screaming and violent trembling all over; single parts seem to be convulsed; spasms of children, preceded by hasty drinking; convulsive twitching, especially after fright or grief (of the nurse); convulsions during dentition, with frothing at the mouth, kicking with the legs; deep sighing and sobbing, with a strange, compressed feeling in the brain; the convulsions commence and terminate with groaning and stretching of the limbs; the paroxysms are accompanied with vomiting; fright with grief may have been the exciting cause; recent cases of epilepsy; convulsions return at the same hour in the daytime or night; silent, stupid state, with jerking of the body, partial spasms of the extremities, one limb, or only certain muscles at a time; emotional epilepsy; lassitude after the fit.

Indigo.—Epilepsy; patient is of exceedingly melancholic (blues) character, tired of life, feels very gloomy; flushes of heat from abdomen to head, with sensation as if the head was tightly bandaged around the forehead; epileptic fit begins with dizziness; epilepsy originating from plexus solaris, or from abdominal ganglia, or from a cold or fright.

Ipecac.—Much nausea and vomiting, either before or during a spasm; the child is spasmodically drawn in some direction; body rigid, stretched out, followed by spasmodic jerking of the arms; convulsions from indigestible food or from suppressed eruption; one constant sensation of nausea all the time, with occasional convulsions; such symptoms—convulsions characterized by continuous nausea—are always relieved by Ipecac alone.

Kali-brom.—Mental hebetude, slowness of expression, failure of memory; confusion and heat of the head, great vertigo; dull, stupefied expression; the same languor in extremities, in fact the whole mind and body given up to lassitude, but nowhere convulsions.

Kali-carb.—The spasm seems to be relieved or to pass off by frequent eructations.

Kreosote.—Convulsion from the swelling of a gum over a tooth which is not quite through; great restlessness; wants to be in motion all the time, and screams the whole night; bronchial irritation from dentition; the teeth look black and decay as fast as they appear; otitis.

Lachesis.—The convulsions are particularly violent in the lower limbs, with coldness of the feet, stretching backward of the body and screaming; epileptic convulsion characterized by cries, falling down unconsciously, foam at the mouth, sudden and forcible protrusion of the tongue; vertigo, heavy and painful head; palpitation of heart; left side chiefly affected; onanism or excessive sexual desire the cause of the disease.

Laurocerasus.—Much gasping for breath before, during, or after a spasm, with bluish tint of the skin; after fright; emaciation; she is conscious of a shock passing through her whole body before the spasm [Hell.].

Lycopodium.—Spasms from incarcerated flatus, with screaming, foaming at the mouth, throwing the arms about, unconscious.

Moschus.—Convulsions from uræmic poisoning.

Nux-mosch.—Convulsive motion of the head from behind forward; hysterical eclampsia in women who easily faint; drowsy before and after the spasm.

Nux-vomica.—Convulsions in the child from indigestion, especially through the high living of the nurse, from emotions in the nurse, as anger; the spasms begin with an aura in the epigastrium; spasms renewed by the least touch, followed by deep sleep; spasms renewed whenever the feet are touched; great torpor of the intestinal canal; in persons who are of an irritable disposition and in those who are accustomed to wines and high living generally, and who lead a sedentary life; spinal epilepsy, with opisthotonos; trembling or convulsive twitching of the limbs; involuntary defecation and urination; rigidity of the limbs; pressure on solar plexus renews the attack.

Œnanthe-crocata.—Epileptiform convulsions followed by deep sleep or coma; convulsions with vertigo, madness, nausea, vomiting; unconsciousness, eyeballs turned up, pupils dilated, lockjaw; convulsions with deathlike syncope; epilepsia nocturna.

OPIUM.—Spasms from fright, anger [nurse]; in children from the approach of strangers; in new-born babes, screaming before or during the spasms; after attack, deep sleep; stupor between spasms; sopor with stertorous respiration; the stertorous respiration continues constantly from one spasm till the next, and so on; incoherent wanderings and convulsive rigidity of the body, with redness, swelling, and heat of the face; hot perspiration and insensible pupils; suppression of the pains of labor may have been the proximate cause; nocturnal epilepsy; continued stertorous breathing; respiration deep, unequal; cyanotic face, or red, bloated, distorted; deep, comatose sleep; suffocative paroxysms during convulsive state.

PHOSPHORUS.—Previous to the convulsion a sensation of heat rushes up the back into the head; this was several times perceived as a forerunner of the convulsion.

PLUMBUM.—Epilepsy; heaviness and numbness of the legs before the spell; swollen tongue; after the fit consciousness returns only slowly and symptoms of paralysis remain; chronic cases with earthy color of face, stupor and debility after fit; periodicity.

PULSATILLA.—The countenance is cold, clammy, and pale; loss of consciousness and of motion; stertorous breathing and full pulse; the labor-pains are deficient, irregular or sluggish, otherwise she is in good condition; mild and tearful; the patient demands fresh air.

SECALE-COR.—Twitching of single muscles; twisting of the head to and fro; contortions of the hands and feet; labored and anxious respiration; in scrawny, illy-nourished women, with too feeble labor-pains; "ergotismus convulsivus."

SILICEA.—Spasms which return at the change of the moon or at night; convulsions after vaccination; attacks preceded by coldness of the left side, shaking and twisting of the left arm; nocturnal epilepsy, especially about the time of the new moon; chronic cases [after Calc.]; before the attack; feeling of great coldness of left side of the body, shaking of the left arm; slumber with starting; the spasms spread, undulating from the solar plexus up toward the brain; violent screaming, groaning, tears drop out of his eyes, foam before the mouth; afterward, warm perspiration, slumber, paralysis of the right side; exalted susceptibility to nervous stimuli, with an exhausted condition of the nerves; abdominal epilepsy.

STANNUM.—Spasms during dentition, with worm symptoms, more excitability, more disturbance of the brain and more fear than Cina; helminthiasis of genital orgasm; epilepsy with tossing of the limbs; clenching of the thumbs; pale face, opisthotonis, unconsciousness.

STRAMONIUM.—Suppression of an eruption, or the exanthem fails to come out; the child is afraid and shrinks back from objects on first seeing them; opisthotonic convulsions from bright, dazzling objects, water, or touch; abdomen puffed; body very hot; spasms continually change character; frightened appearance before and after the convulsions commence; sardonic grin; stammering or loss of speech; loss of consciousness and sensibility; frightful visions; laughter, singing; attempts to escape; the fits are renewed by the sight of brilliant objects, and sometimes by contact; epileptiform spasms; thrusting the head continually in quick succession to the right; continual rotary motion of the left arm; pains in the pit of the stomach; obstinate constipation; deep, snoring sleep; risus sardonicus; pale, worn-out appearance, with a stupid look; afraid of being alone; convulsions affecting the upper more than the lower extremities; also isolated groups of muscles.

SULPHUR.—Whenever some dyscrasia lurks in the system, or its outward symptoms were suppressed; chronic epilepsy; before the spell; crawling and running as from a mouse down the back and arms, or up the leg to right side of the abdomen; after the convulsions, soporous sleep and great exhaustion.

TARENTULA.—Hysteric-epilepsy; sensation of dizziness before the fit, followed by convulsions and great præcordial anguish.

TART.-EMET.—Spasms from repelled eruptions, with paleness of the skin and much difficulty of breathing; great prostration and faintness.

TEREBINTHINA.—Dentition accompanied by suppression of urine and convulsions; child is wakeful at night, screaming as if frightened, has a staring look, clenches his fingers; twitchings in different parts of the body; picking of nose; dry, short cough, aching in limbs and head; burning soreness and interstitial soreness of gums; otitis infantilis.

VERATRUM-ALB.—Convulsions of children, with pale face and cold sweat on the forehead; cough before or after the spasm; trembling all over.

VERATRUM VIRIDE.—Eclampsia from emotional causes; great activity of the arterial system; convulsions and mania, which even keeps on after the cessation of the spasms; face flushed, pulse wiry, thirst.

VISCUM-ALB.—Epilepsy, with constant vertigo, even when in bed; feeling as if the whole vault of the skull would be raised up; muscles of the face in constant agitation.

ZINCUM.—Twitching in various muscles; the whole body of the child jerks during sleep; the child is cross before the attack; body hot; restless at night; fidgety feet; right side twitches; pale children during teething; after the disappearance of old eruptions; coma from cerebral exhaustion; loss of sensation of the whole body; mania from mental excitement; somnambulism; Zinc. has been known to cure obstinate puerperal convulsions after Phosphorus, apparently indicated, had failed; cerebral epilepsy; symptoms felt mostly during rest; aggravation after dinner and toward evening; twitching in various muscles; the whole body jerks during sleep.

ZIZEA AUREA.—Spasmodic movements of the muscles of the face and extremities; epilepsy.

---

DISCUSSION.—Dr. Ballard—I want to speak of one case of convulsions, in which I followed the remedy as closely as could be, by study in the sick-room and in my office. The child's first convulsion came on Tuesday morning, and I was soon there. I noticed at the termination of the convulsion there was a straining, the face turning red, just as a child would do straining hard for stool. When the convulsions came the eyes were rolled up and there was some twitching of the mouth. Various remedies were tried, yet no relief. There was that only symptom of straining. It went on until Friday morning, when I gave the child Nux $^{200}$, and left, saying that "if there is no improvement in two hours, give this powder." The powder I left was a dose of Belladonna. On my return I found that the powder had been given. "Why did you give the second powder?" "I misunderstood you, I thought necessary to give it." Anyway, I did not notice so much of that straining. The convulsions were about as bad as before. I gave another dose of Nux and the convulsions ceased almost immediately, and the symptoms ceased with them entirely. Has any one noticed that symptom under Nux.? Following the convulsions there came a large number of boils. Another case: After the delivery of twins the mother at once went into convulsions, and the lochia ceased. The urine was suppressed. The convulsions would be preceded or accompanied by grunting, twitching of the face, throwing back of the eyes, and then finally closing them. The twitching would last for a half a minute or so, and then a sinking down and quiet for a few seconds, and then would start in breathing with the throat extended beyond the jaw; the face bluish or purplish, collar covered with sweat of a warm character; frothing at the mouth and every appearance of Opium. Chamomilla had been given and hot compresses. When I saw this condition, I gave Opium. There was no help from it at all; the convulsions continued. The woman lay on her back, with the left leg fixed, and the other leg thrown over and kept in constant motion over the opposite knee. During the convulsions there was an emission of urine which had a strong urinal odor. After giving Belladonna and seeing no change from this constant motion, I gave a dose of Argentum-nit. I then noticed for the first time the most marked motion of the *alæ nasi*, which was soon over. As soon as that was over I gave a dose of Lycopodium. The symptoms were slightly relieved; I thought the motion of the *alæ nasi* was less prominent, and the convulsion was not as long. The motion or restlessness afterward continued the same. When the third convulsion came on, there was no indication of that. That was the last, and recovery commenced, and she made an immediate and good recovery.

Dr. Emory—We must have our *Materia Medica* in our minds or some very easy reference to it, and I think that paper completed and published would be of very valuable assistance to many of us. There are some remedies which we consider useful in these cases which Dr. Holmes has not mentioned. There is one symptom which Hering gives which I did not notice, and that is "spasms occurring at the time of the menses;" either where the menses do not occur at the proper time, or do occur.

Dr. Custis—I think one reason you do not get the prompt action of the remedy, is often on account of the nature of the convulsion, which is due to the frothing at the mouth. If there is anything that will interfere with the absorption of medicine it is that frothing. I had a case a few weeks ago of a little boy suddenly attacked by convulsions, the last one of which lasted over three hours. In spite of all efforts I could make I could not administer a remedy, as there was a complete locking of the jaws and a constant frothing at the mouth. I felt sure that I had the right remedy, but I could not get any results, as I could not administer it properly. I thought of a hypodermic syringe, and this is the only time I ever resorted to that; but I shall remember to do it again.

Dr. Wesselhœft—I think Dr. Custis would have had the same result if he had given that remedy by olfaction.

Dr. Custis—I would like to ask Dr. Ballard if the urine of the patient had been examined.

Dr. Ballard—I examined it and found no albumen in it, although there had been enormous swelling of the legs all through the pregnancy. This disappeared a few days before the confinement. A few months before the confinement the patient was melancholy.

Dr. Holmes—Two years ago this month I was called to see the worst case of convulsions in an infant I ever saw. It was just before I came to attend the American Institute at Saratoga, and it was before I knew much about Homœopathy. The little fellow was two weeks old. He went into a convulsion at 12 o'clock at night, and for almost twenty-four hours that child laid in convulsions most of the time. At times the child would apparently cease living; pulsation down to twenty a minute, respiration would cease. I used artificial respiration probably twenty times. My first prescription was Nux-vomica. I went on with my Nux-vomica study, and the more I studied the case the more I was convinced that Nux-vomica was the remedy, I then gave the sixth and nothing else, and that little fellow to-day is one of the stoutest, ruddiest little boys you ever saw. In the course of twelve hours after giving the remedy there were no more spasms. This shows how some of those terrible cases can get well if you give the right remedy.—*Clinical Bureau, I. H. A.*

